# Landscape of germline genetic alterations among non-western young male patients with cancer. Findings from The Jordanian exploratory cancer genetics study

**DOI:** 10.3389/fonc.2026.1867571

**Published:** 2026-07-29

**Authors:** Hikmat Abdel-Razeq, Hira Bani Hani, Seif Alafeef, Faris Tamimi, Areej Al-Atary, Lulwa El Saket, Zeidan Zeidan, Malek Horani, Hala Abu-Jaish, Baha’ Sharaf, Suleiman Mahafdah, Amerah Abdelqader

**Affiliations:** 1 Section of Hematology and Medical Oncology, Department of Internal Medicine, King Hussein Cancer Centre., Amman, Jordan; 2 School of Medicine, the University of Jordan., Amman, Jordan; 3 Department of Surgery, Jordanian Royal Medical Services, Amman, Jordan

**Keywords:** BRCA, germline genetic testing, hereditary cancer predisposition syndromes, lynch, male

## Abstract

Early-onset cancers in males may indicate hereditary predisposition, yet real-world data on germline testing across diverse tumor types are limited. We evaluated the prevalence and spectrum of germline pathogenic/likely pathogenic (P/LP) variants in males diagnosed with cancer at ≤50 years. Consecutive male patients aged ≤50 years with newly diagnosed solid tumors enrolled in the Jordanian Exploratory Cancer Genetics (Jo-ECAG) program at the King Hussein Cancer Center underwent multigene panel germline testing irrespective of cancer type, stage, or family history. Patients were classified according to National Comprehensive Cancer Network (NCCN) testing criteria. P/LP and variant of uncertain significance (VUS) rates were analyzed by age, cancer type, and eligibility status. Among 652 patients (median age, 42 years), colorectal cancer predominated (48.0%), followed by gastric (6.7%), pancreatic (6.4%), and testicular (6.0%) cancers. Overall, 90 patients (13.8%) harbored P/LP variants and 216 (33.1%) had VUS. P/LP detection was higher among patients meeting NCCN criteria compared with those who did not (16.5% vs. 4.7%, p<0.001). Younger age was associated with higher yield (26.3% in ages 18–30 vs. 9.7% in 41–50 years, p<0.001). Colorectal cancer accounted for most pathogenic findings. Frequently altered genes included *APC, MLH1, MSH2, MUTYH, BRCA2*, and *TP53*; no BRCA1 P/LP variants were identified. Germline P/LP variants are common in young male patients with cancer, particularly at younger ages and among those meeting testing criteria. These findings support broader implementation of germline testing to inform management and familial risk assessment.

## 1 Introduction

Hereditary cancer predisposition syndromes represent a clinically significant subset of malignancies, with germline pathogenic or likely pathogenic (P/LP) variants estimated to account for approximately 5-10% of all cancers overall and a substantially higher proportion among patients diagnosed at a young age (1–4). These inherited variants typically involve genes responsible for DNA damage repair (5), genomic integrity, and cell-cycle regulation, resulting in increased lifetime cancer risk, earlier age of onset, aggressive disease biology, and a propensity for multiple primary malignancies (6–8).

Early-onset cancer has long been recognized as a cardinal feature suggestive of an underlying hereditary etiology, even in the absence of a striking family history. Population-based sequencing studies and large institutional cohorts have consistently demonstrated that younger age at diagnosis is among the strongest independent predictors of identifying a germline pathogenic variant (9). This association is particularly well established in colorectal cancer (10), pancreatic cancer (11), prostate cancer (12), sarcomas (13), and male breast cancer, where germline findings have direct implications for surveillance, therapeutic decision-making, and familial risk assessment (9, 14).

Over the past decade, the widespread adoption of next-generation sequencing (NGS)-based multigene panel testing has transformed the landscape of hereditary cancer evaluation (15, 16). Rather than focusing on single-gene testing driven by classic phenotypes, contemporary practice increasingly favors multigene panels capable of simultaneously interrogating dozens of cancer susceptibility genes (17–20). This evolution has led to improved detection of actionable germline variants but has also introduced challenges, including high rates of variants of uncertain significance (VUS) and uncertainty regarding optimal patient selection for testing (10).

Professional societies such as the National Comprehensive Cancer Network (NCCN) (21, 22). and the American Society of Clinical Oncology (ASCO) (23–25) have responded to these developments by progressively expanding recommendations for germline genetic testing across tumor types and age groups. Notably, several malignancies historically considered sporadic, such as pancreatic, prostate, and gastric cancers, are now recognized to have meaningful hereditary contributions, particularly when diagnosed at younger ages (26, 27).

Despite these advances, the majority of available germline testing data remain disproportionately focused on female-predominant cancers, especially breast and ovarian cancer (28–30) This emphasis has resulted in a relative underrepresentation of men in hereditary cancer research, even though men may carry and transmit pathogenic variants at similar rates. Moreover, several cancers that disproportionately affect men, including colorectal, testicular (31), pancreatic, prostate, renal, and head and neck cancers, have established or emerging hereditary associations that may be overlooked in routine practice.

Under-recognition of inherited cancer susceptibility in men carries important clinical consequences. Missed germline diagnoses may delay intensified screening for secondary malignancies, limit opportunities for risk-reducing interventions, and prevent cascade testing of at-risk relatives. Furthermore, germline findings increasingly influence therapeutic decision-making. Pathogenic variants in homologous recombination repair genes may confer sensitivity to poly (ADP-ribose) polymerase (PARP) inhibitors in prostate and pancreatic cancers, while mismatch repair deficiency predicts responsiveness to immune checkpoint inhibition across tumor types (32–36).

Real-world data describing the prevalence, age-specific trends, and gene-level spectrum of germline alterations among young male patients with cancer remain limited. Prior studies have either focused on single tumor types or included relatively small numbers of male patients, limiting generalizability (37–40). Additionally, the performance of current guideline-based testing criteria in young male populations has not been comprehensively evaluated.

To address these gaps, we conducted a large retrospective analysis of germline multigene panel testing results in males diagnosed with cancer at age 50 years or younger. Our objectives were to determine the prevalence of germline P/LP variants, evaluate differences in testing yield by age and cancer type, assess the effectiveness of guideline-based testing criteria, and characterize the spectrum of implicated cancer susceptibility genes. By focusing on a real-world cohort spanning diverse malignancies, this study aims to inform future strategies for optimizing hereditary cancer detection in young men.

## 2 Methods

### 2.1 Study design and patient population

This retrospective observational study evaluated germline genetic testing outcomes among male patients diagnosed with cancer at age 50 years or younger between April 2021 and August 2023. All patients included had a confirmed malignant diagnosis and underwent clinical germline genetic testing using multigene NGS panels as part of routine oncologic care or as part of an ongoing clinical trial evaluating the concept of universal germline genetic testing of all patients with solid tumors (41). Patients with incomplete demographic, clinical, or genetic testing data were excluded from analysis. Age at diagnosis was analyzed both as a continuous variable and as predefined categorical groups (18-30, 31-40, and 41–50 years) to evaluate age-related trends in germline variant detection.

### 2.2 Clinical data collection

Clinical and demographic data were abstracted from institutional genetic testing and patients’ electronic medical records. Variables collected included age at diagnosis, primary cancer type, and eligibility for germline genetic testing according to contemporaneous professional guidelines. Cancer diagnoses were categorized by primary tumor site using standard oncologic definitions. Eligibility for germline genetic testing was determined based on NCCN and ASCO criteria applicable at the time of testing, incorporating factors such as early age at diagnosis, tumor type, multiple primary malignancies, and features suggestive of a hereditary cancer syndrome.

### 2.3 Germline genetic testing procedures

All germline testing was performed in Clinical Laboratory Improvement Amendments (CLIA)-certified laboratories using NGS-based multigene panels. Panels included genes associated with hereditary cancer predisposition, encompassing tumor suppressor genes, DNA mismatch repair genes, and genes involved in homologous recombination repair, base excision repair, and genomic stability. Panel composition evolved over the study period; however, all panel versions consistently included the major clinically actionable hereditary cancer susceptibility genes, including *APC, BRCA1, BRCA2, MLH1, MSH2, MSH6, PMS2, EPCAM, TP53, CHEK2, ATM, PALB2*, and *MUTYH*. Testing evaluated single-nucleotide variants and small insertions/deletions. Copy number variant analysis was performed when available as part of the testing platform. The specific gene content varied slightly over time as panels evolved, but core hereditary cancer genes were consistently included.

### 2.4 Variant interpretation and classification

Identified germline variants were classified by the testing laboratories according to American College of Medical Genetics and Genomics and Association for Molecular Pathology (ACMG/AMP) standards. Variants were categorized as pathogenic, likely pathogenic, variant of uncertain significance (VUS), likely benign, or benign. In addition, variants reported by the testing laboratories as increased-risk alleles (low-penetrance risk alleles) were considered a separate classification and were not included in the VUS category.

For analytic purposes, P/LP variants were grouped together as clinically actionable findings. Variants classified as increased-risk alleles (e.g., monoallelic MUTYH) were documented separately. Patients harboring more than one germline alteration were identified, including those with dual pathogenic variants.

### 2.5 Outcomes and statistical analysis

The primary outcome was the prevalence of P/LP germline variants in the overall cohort. Secondary outcomes included differences in P/LP and VUS detection rates by age group, cancer type, and testing eligibility status, as well as the distribution of implicated cancer susceptibility genes.

Descriptive statistics were used to summarize patient characteristics and genetic testing outcomes. Categorical variables were compared using chi-square or Fisher’s exact tests, as appropriate. Two-sided P values <0.05 were considered statistically significant. All analyses were exploratory and descriptive in nature.

### 2.6 Ethical considerations

This study was conducted in accordance with the Declaration of Helsinki. Given the retrospective design and use of de-identified data. The study was approved by institutional review board (IRB) at King Hussein Cancer Center, however, informed consent was waived in accordance with institutional policy.

## 3 Results

### 3.1 Patient characteristics

**Figure 1:**
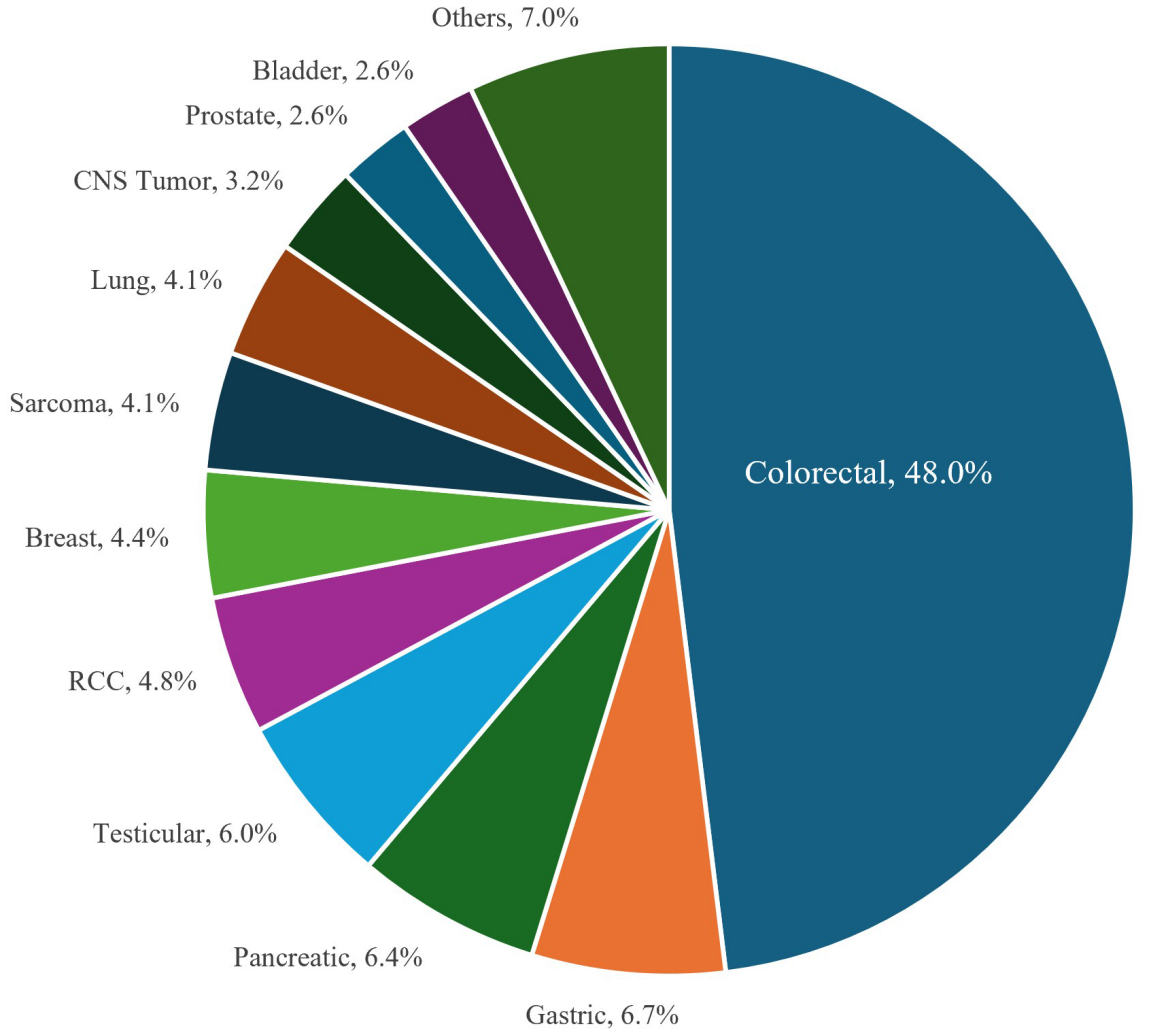
Common primary cancer sites included in the study.

A total of 652 male patients diagnosed with cancer at age 50 years or younger were included in the analysis. The median age at diagnosis was 42 years (range, 18-50). Most patients were diagnosed between ages 41 and 50 years (n = 370, 56.7%), followed by those aged 31–40 years (n = 202, 30.9%), while a smaller subset was diagnosed between ages 18 and 30 years (n = 80, 12.3%). Colorectal cancer was the most common malignancy in the cohort, accounting for nearly half of all patients (n = 313, 48.0%). Other frequently observed cancer types included gastric cancer (n = 44, 6.7%), pancreatic cancer (n = 42, 6.4%), testicular cancer (n = 39, 6.0%) and renal cell carcinoma (n = 31, 4.8%). Less common malignancies included male breast cancer, lung cancer, sarcoma, central nervous system (CNS) tumors, prostate, bladder, head and neck, thyroid, melanoma and esophageal cancer, **Figure 1**.

### 3.2 Overall germline genetic testing outcomes

Across the entire cohort, 90 patients (13.8%) were found to harbor pathogenic or P/LP germline variants. An additional 216 patients (33.1%) had at least one VUS. The remaining patients had no clinically reportable germline findings. Thirteen patients (2.0%) carried more than one germline alteration; among these, five patients had two pathogenic variants, while eight had one pathogenic variant and one increased-risk allele.

### 3.3 Germline testing eligibility and diagnostic yield

**Figure 2:**
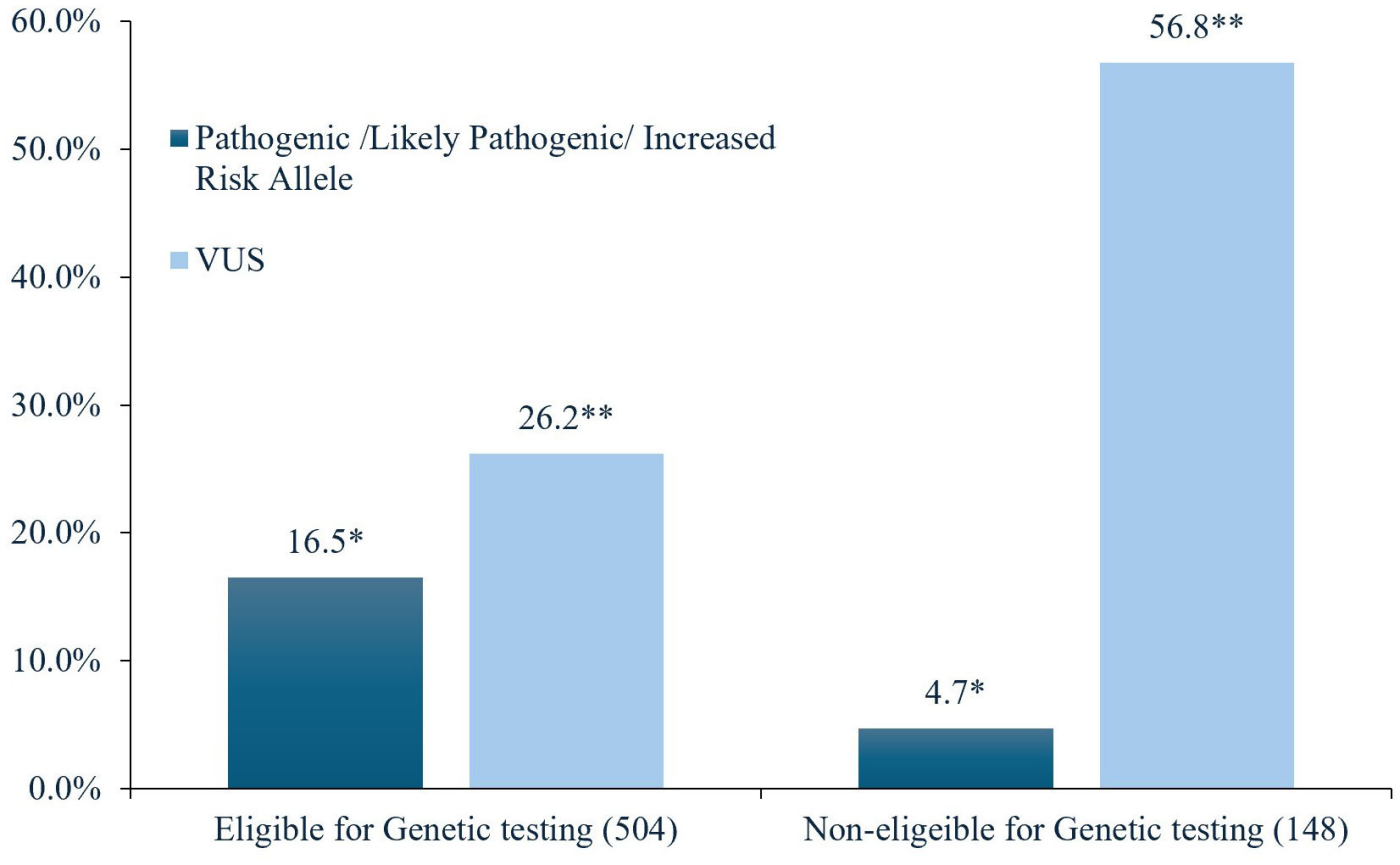
Results of germline genetic testing according to testing eligibility.

Of the 652 patients, 504 (77.3%) met established NCCN and ASCO criteria for germline genetic testing at the time of evaluation, while 148 (22.7%) did not meet guideline-based eligibility criteria and were tested as part of clinical trial evaluating the role of universal germline genetic testing for patients with solid tumors.42 Patients who met testing criteria demonstrated a significantly higher prevalence of pathogenic findings compared with those who did not (16.5% vs. 4.7%, p < 0.001). Variants of uncertain significance were also differentially distributed according to eligibility status. Among eligible patients, 132 (26.2%) had VUS, whereas more than half of non-eligible patients harbored VUS (n = 84, 56.8%; p < 0.001), **Figure 2**.

### 3.4 Age-specific germline variant detection

**Figure 3:**
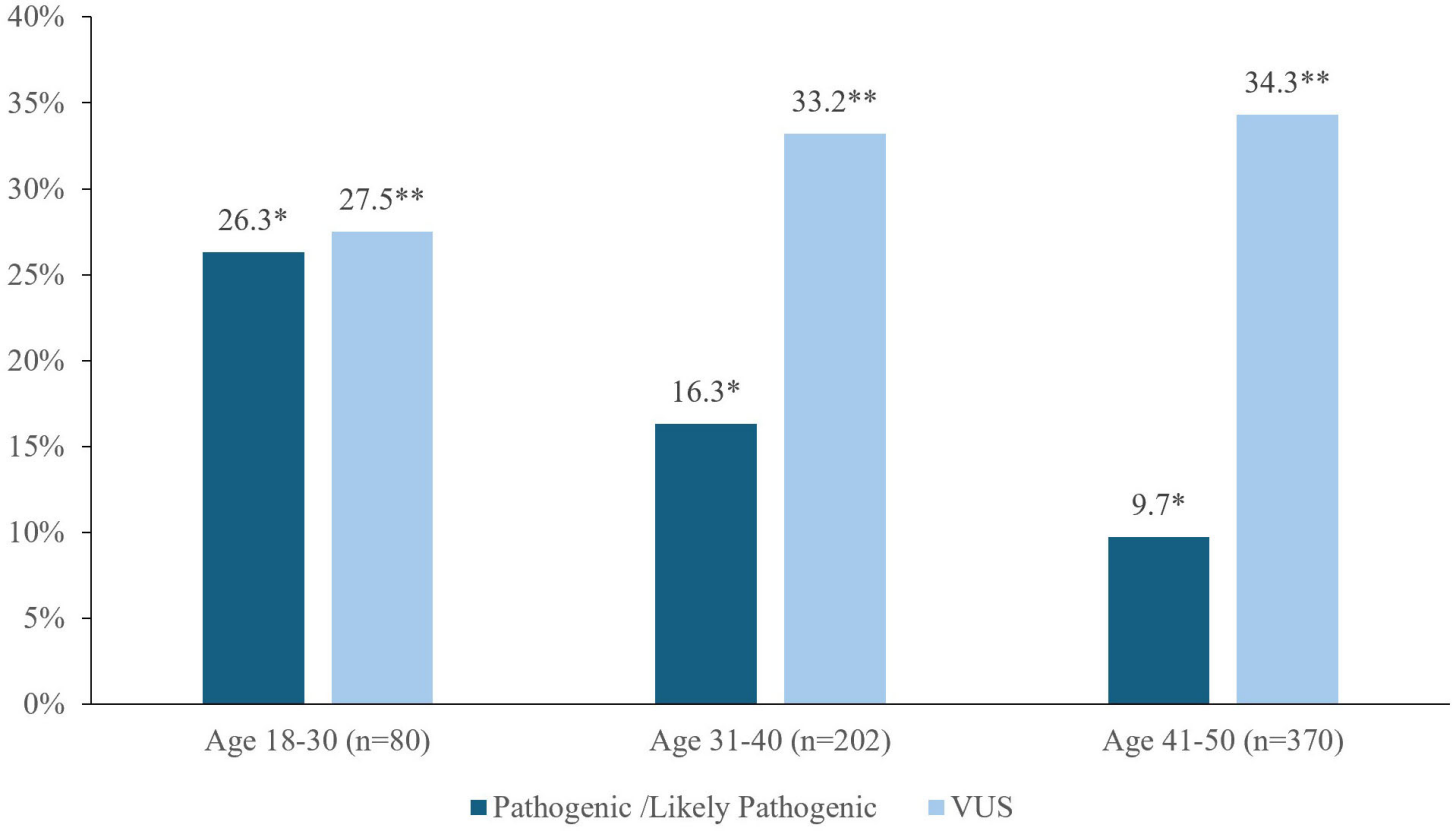
Results of germline genetic testing according to age group. (*) and (**) refers to the p-value indicated immediately below the table.

A strong inverse association was observed between age at cancer diagnosis and the prevalence of pathogenic germline variants. Patients diagnosed between ages 18 and 30 years had the highest rate of P/LP variants, with more than one-quarter affected (21 of 80 patients, 26.3%). In comparison, pathogenic variants were detected in 16.3% of patients aged 31–40 years (33 of 202) and in only 9.7% of those aged 41–50 years (36 of 370). This age-related trend was statistically significant (p < 0.001). In contrast, VUS detection rates were relatively consistent across age groups, occurring in 27.5% of patients aged 18–30 years, 33.2% of those aged 31–40 years, and 34.3% of those aged 41–50 years (p = 0.501), **Figure 3**.

### 3.5 Cancer-type–specific germline findings

**Figure 4:**
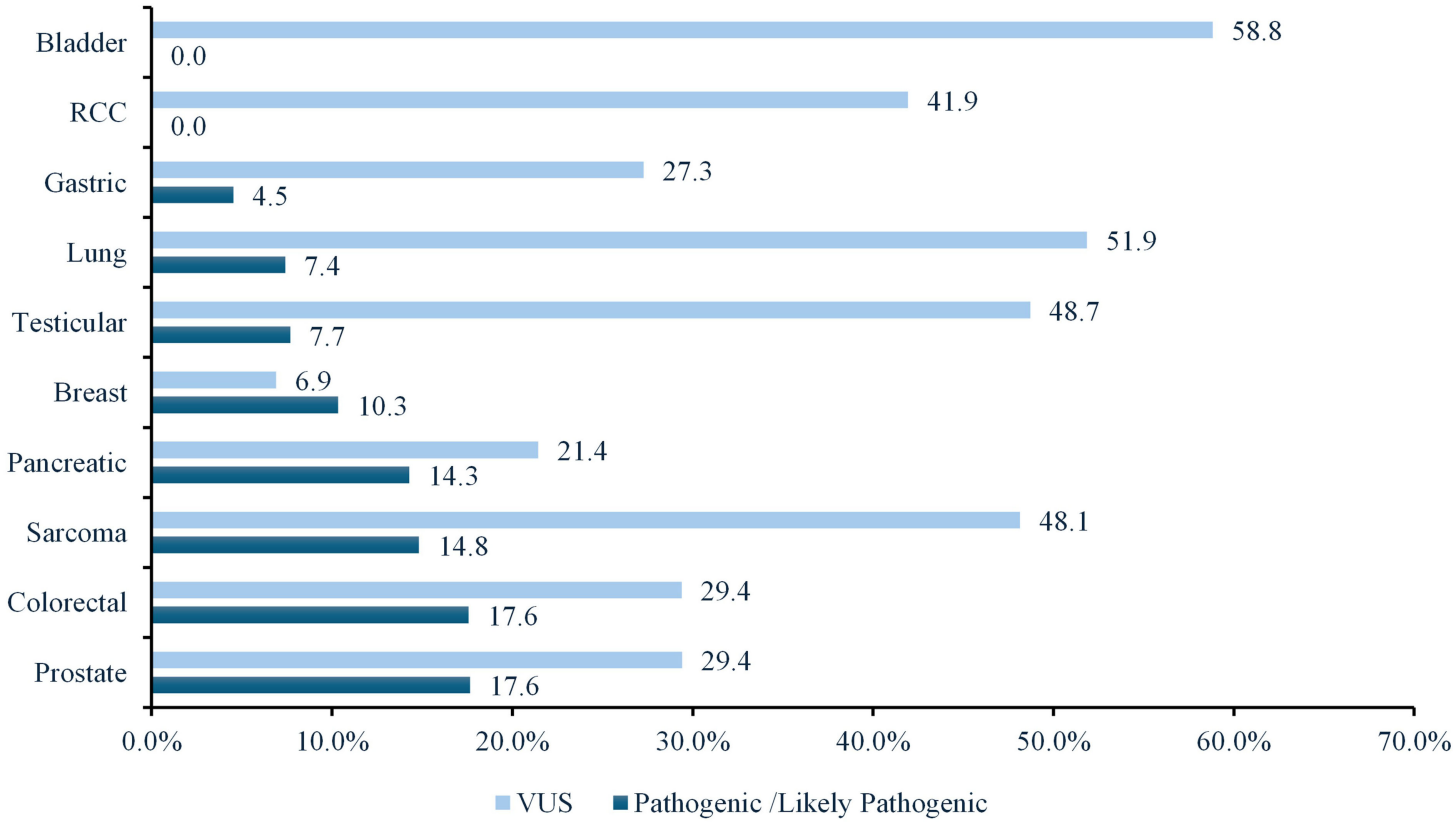
Rates of P/LP and VUS according to primary cancer in percent. RCC, renal cell carcinoma; P/LP, Pathogenic/Likely Pathogenic; VUS, Variants of Uncertained Significance.

The prevalence of pathogenic germline variants varied substantially by cancer type. Among patients with colorectal cancer, 55 individuals (17.6%) harbored P/LP variants, representing the largest absolute contribution of pathogenic findings in the cohort. VUS were identified in an additional 94 colorectal cancer patients (29.4%). Pancreatic cancer demonstrated a relatively high pathogenic yield, with P/LP variants detected in 6 of 42 patients (14.3%). Similar pathogenic rates were observed in sarcoma (4 of 27 patients, 14.8%) and CNS tumors (3 of 21 patients, 14.3%). Prostate cancer also showed a notable pathogenic variant prevalence, with 3 of 17 patients (17.6%) affected. In contrast, certain tumor types, including renal cell carcinoma, bladder cancer, melanoma, and esophageal cancer, showed no pathogenic variants despite high rates of VUS, particularly in bladder and head and neck cancers, where VUS rates exceeded 50%, **Figure 4**.

### 3.6 Spectrum of germline alterations and gene distribution

**Figure 5:**
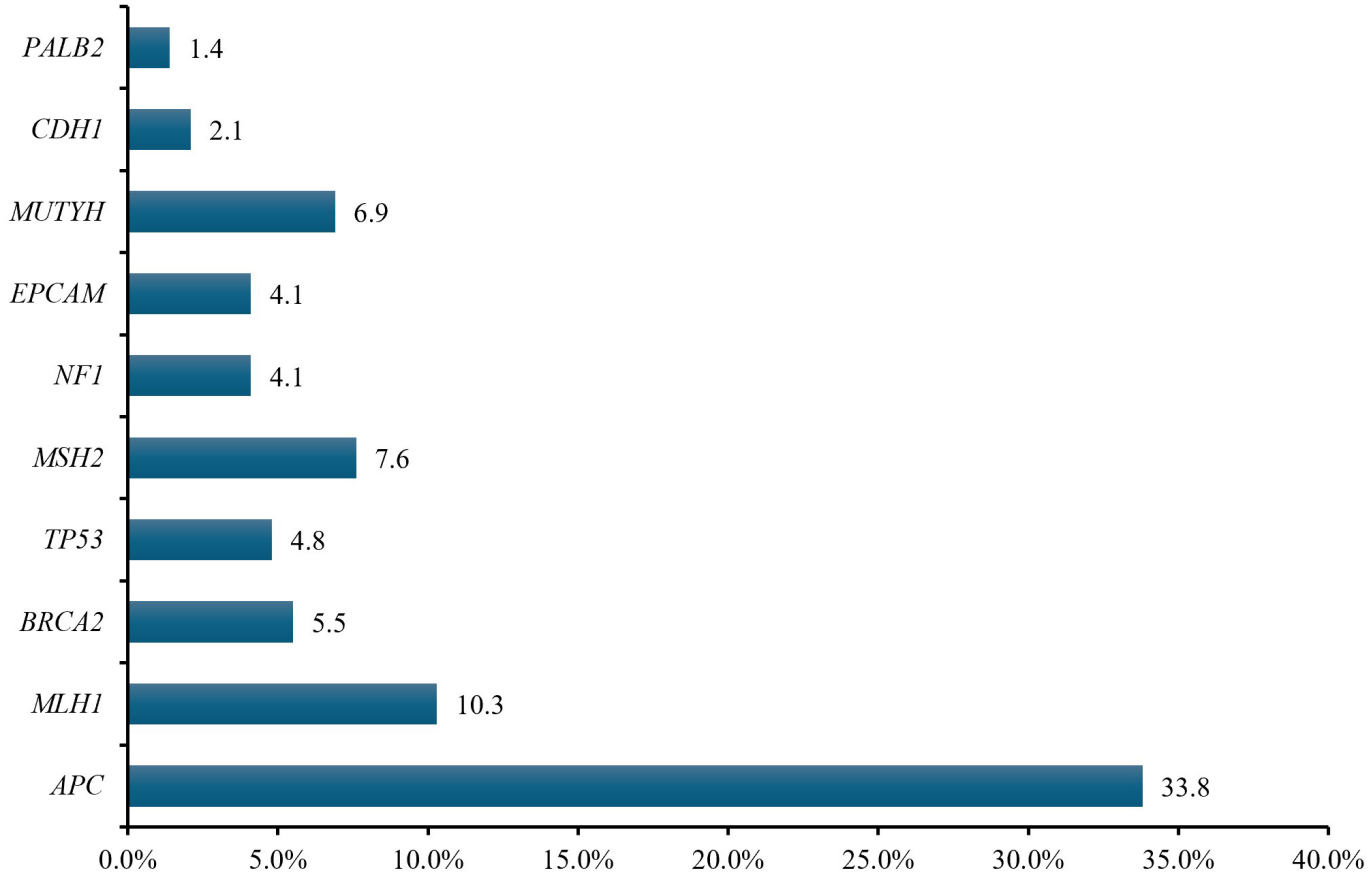
Frequency of specific gene mutation (pathogenic/likely pathogenic).

Across the cohort, a total of 145 pathogenic or increased-risk alleles were identified. The most frequently mutated gene was *APC*, accounting for 49 variants (33.8%). Mismatch repair genes collectively represented a major proportion of pathogenic findings, including *MLH1* (n=15, 10.3%), *MSH2* (n=11, 7.6%), and *EPCAM* (n=6, 4.1%), consistent with Lynch syndrome-associated cancers. Other recurrently affected genes included *MUTYH* (n=10, 6.9%), *BRCA2* (n=8, 5.5%), *TP53* (n=7, 4.8%), *NF1* (n=6, 4.1%), and *CDH1* (n=3, 2.1%), **Figure 5**.

## 4 Discussion

In this large real-world cohort of young male patients diagnosed with cancer at age 50 years or younger, we demonstrate that germline P/LP variants are present in nearly 14% of patients. This prevalence exceeds traditional estimates for unselected cancer populations and underscores the substantial contribution of hereditary cancer predisposition in young men.

A key finding of this study is the strong inverse relationship between age at diagnosis and pathogenic variant detection. More than one-quarter of patients diagnosed before age 30 harbored P/LP variants, compared with fewer than 10% of those diagnosed between ages 41 and 50 years. These findings align with prior reports demonstrating that younger age is among the most powerful predictors of germline pathogenicity across cancer types (37, 43). Our data reinforce the importance of aggressive consideration of germline testing in very young male patients, even in the absence of a striking family history.

The overall prevalence of P/LP germline variants observed in our study (13.8%) is comparable to that reported in several large Western studies evaluating unselected or broadly selected patients with cancer. Universal germline testing studies from North America have generally reported pathogenic variant detection rates ranging from approximately 10% to 18%, depending on patient selection criteria, tumor composition, age distribution, and the multigene panel employed (9). Our findings therefore suggest that hereditary cancer predisposition among young male patients in the Middle East is at least comparable to that observed in Western populations, despite important differences in ethnicity, healthcare systems, referral patterns, and family structures.

While the overall prevalence of pathogenic variants was similar, the distribution of affected genes reflected the composition of our cohort. Consistent with the predominance of early-onset colorectal cancer, *APC* and Lynch syndrome-associated mismatch repair genes accounted for a substantial proportion of pathogenic findings. In contrast, Western pan-cancer cohorts that include larger numbers of breast, ovarian, and prostate cancers frequently report *BRCA1* and *BRCA2* among the most commonly altered genes. The relative scarcity of *BRCA1* pathogenic variants in our cohort likely reflects the exclusively male study population and the relatively small number of male breast cancer cases, rather than true population-specific differences in hereditary breast cancer susceptibility.

Our findings also contribute important data from an understudied population. Most large hereditary cancer sequencing studies have been conducted in North American and European populations, whereas genomic data from Arab and Middle Eastern populations remain comparatively limited. Underrepresentation of these populations has important implications for hereditary cancer research, including limited knowledge of population-specific pathogenic variants and higher rates of variants of uncertain significance resulting from incomplete representation in publicly available genomic databases. Expanding germline testing data from diverse populations is therefore essential to improve variant interpretation, facilitate reclassification of uncertain variants, and ensure equitable implementation of precision oncology worldwide.

Collectively, these findings demonstrate that although the overall burden of hereditary cancer predisposition appears comparable to that reported internationally, the spectrum of associated genes largely reflects the underlying cancer distribution and population characteristics. These observations underscore the importance of interpreting germline testing results within the clinical and epidemiologic context of the population being studied rather than extrapolating directly from predominantly Western cohorts.

Colorectal cancer emerged as the dominant tumor type associated with pathogenic germline findings, accounting for the largest absolute number of P/LP variants. This observation is consistent with previous studies demonstrating a high prevalence of hereditary cancer syndromes, particularly Lynch syndrome and familial adenomatous polyposis, among young patients with colorectal cancer (44–47). The predominance of APC and mismatch repair gene mutations in our cohort further emphasizes the critical role of germline testing in early-onset colorectal cancer in men.

An important consideration when interpreting our findings is that colorectal cancer represented nearly half of the study cohort. Consequently, the overall prevalence of pathogenic germline variants and the predominance of APC and mismatch repair gene alterations are likely influenced by the high representation of this tumor type, which is well recognized for its strong hereditary component in early-onset disease. Accordingly, our findings should not be interpreted as reflecting an equal distribution of hereditary susceptibility genes across all cancer types. Rather, this cohort represents the real-world spectrum of consecutive young male patients undergoing germline genetic testing at our institution. Nevertheless, pathogenic variants were identified across multiple non-colorectal malignancies, including pancreatic, prostate, sarcoma, and central nervous system tumors, supporting the broader clinical utility of multigene germline testing in selected young male patients with diverse cancers.

Notably, pathogenic variants were also identified across a broad spectrum of malignancies, including pancreatic, prostate, sarcoma, and central nervous system tumors. These findings support expanding the conceptual framework of hereditary cancer beyond traditional phenotype-driven models and highlight the value of multigene panel testing in capturing clinically relevant variants across diverse tumor types.

Patients meeting NCCN and ASCO testing criteria demonstrated a significantly higher yield of pathogenic variants compared with those who did not, validating the effectiveness of guideline-based approaches. However, the detection of P/LP variants among patients who did not meet testing criteria suggests that current guidelines may still miss a subset of hereditary cases, particularly among younger males with atypical presentations. These findings contribute to the ongoing debate regarding age-based or universal germline testing strategies in selected populations (9, 42, 48, 49). While our data support continued evaluation of expanded testing approaches, our study was not designed to determine the clinical utility or cost-effectiveness of universal germline testing, and prospective studies are warranted before broad recommendations can be made.

The high prevalence of VUS observed across cancer types highlights persistent challenges in variant interpretation, particularly in underrepresented populations and rare malignancies (28, 50). While VUS findings should not guide clinical management, their frequency underscores the need for continued data sharing, functional validation, greater inclusion of diverse populations in genomic studies, and ongoing variant reclassification efforts (51, 52).

From a clinical perspective, the implications of germline testing in young men extend beyond risk assessment. Pathogenic variants increasingly inform therapeutic decision-making, including eligibility for PARP inhibitors in BRCA-associated cancers and immune checkpoint inhibitors in mismatch repair–deficient tumors (53, 54). Early identification of hereditary cancer syndromes also enables cascade testing, allowing at-risk relatives to pursue targeted surveillance and prevention strategies (28).

This study has limitations inherent to its retrospective design. Family history data were not uniformly available, and gene panel composition varied over time. Additionally, outcomes such as treatment response and survival were not assessed. Nevertheless, the large sample size, real-world setting, and inclusion of diverse malignancies strengthen the generalizability of our findings.

In conclusion, our findings provide one of the largest descriptions of germline genetic alterations among young male patients with cancer from the Middle East and demonstrate that, while the overall prevalence of pathogenic variants is comparable to that reported in Western populations, the spectrum of affected genes reflects regional tumor distribution and population characteristics (55).

## Data Availability

The raw data supporting the conclusions of this article will be made available by the authors, without undue reservation.
